# Ten Simple Rules for Establishing International Research Collaborations

**DOI:** 10.1371/journal.pcbi.1004311

**Published:** 2015-10-08

**Authors:** Richard de Grijs

**Affiliations:** 1 Kavli Institute for Astronomy and Astrophysics, Peking University, Beijing, China; 2 East Asian Regional Office of Astronomy for Development (EA-ROAD), International Astronomical Union; Accelrys, UNITED STATES

Successful modern research collaborations increasingly include scientists based in different countries. This is partially driven by the need to engage with interdisciplinary science, access innovative approaches to problem solving, and acquire expertise beyond that which your own research group covers. It is also a great way to establish a worldwide network of colleagues with a variety of backgrounds—scientific, cultural, or otherwise. While international collaborations can be very rewarding, both professionally and from a personal perspective, they come with distinct difficulties and pitfalls that one should be aware of a priori. Nevertheless, cultivating an acute awareness of these issues will likely offer rich returns to internationally minded scientists, given that international research collaborations continue to expand, and many are now being established beyond the traditional power players, the North American and European research communities [1].

## Rule 1: Clarify Why You Might Want to Start an International Research Collaboration

Much has been written about the need for interdisciplinarity and cross-fertilization of ideas and methodologies in contemporary science [2]. Although many of the motivations as to why one might want to reach out to colleagues for help and assistance are equally valid for domestic as for international collaborations, pursuing this goal in an international context requires one to consider aspects that may not be as important in a domestic context. You may wonder why there is a need for international collaborations, particularly in fields in which the geographical location of the research project does not play an important role. Indeed, many research communities are split along national boundaries, if not formally then often in practice, as dictated by the strategic goals of the main national funding agencies. Foreign research groups may therefore offer access to complementary perspectives and insights, experience, and skills. Different national research priorities may also have given rise to country-specific differences in terms of the availability of resources or equipment beyond what you can access locally, regionally, or even domestically. International collaborations are indeed essential to tackling the grand challenges of our time [1].

The need to establish international collaborations may, in particular, apply in the context of sharing students, given the enormous differences in student numbers among the different national research communities. It is usually considered professionally advantageous to spend some time abroad during the early stages of a research career (when funding for such exchanges is fairly easily obtained), thus making exchanges of students and junior scientists a particularly interesting vehicle by which to establish international research collaborations. And since current technological advances facilitate easy communication, geographically distributed teams are not necessarily at a disadvantage.

In addition, many international collaborations are established following chance meetings between like-minded individuals, often at scientific meetings. Sharing your research progress with international collaborators with whom you have built up a personal relationship can indeed be very rewarding. In addition to pursuing quality research, the non-tangible benefits may include the development of a widening perspective and learning about different cultural aspects. You may make friends for life, who you will continue to encounter at meetings throughout your career.

## Rule 2: Consider the Characteristics Your International Collaborator Must Have

In addition to considering your motivation to establish an international research collaboration, you should also clarify the desired characteristics of your potential collaborator. This is particularly important if cultural differences or a language barrier may make communication more challenging. In this context, it is interesting to note that even today, many large international networks are split along common-language lines [1]. Consider asking local, trusted colleagues whether they could provide insights as regards your potential collaborator’s reputation of reliability. Does he maintain open communication channels, is she responsive, and do they usually meet important deadlines? Perhaps unsurprisingly, the interpretation of the strictness of internal deadlines may be culturally dependent and, therefore, this aspect could be particularly challenging. Cultural differences are real. They can enrich the collaboration but also cause friction. So, how can we best approach any negative fallout from culturally diverse research collaborations? Two key aspects come to mind: maintain an open mind as well as a flexible attitude. Be aware of the most pertinent cultural differences between researchers hailing from different countries. You do not need to do extensive research into your potential international collaborator’s cultural background, but keeping an open mind, being prepared to respond flexibly, and having a generally heightened awareness of cultural issues will go a long way to resolving the inevitable misunderstandings you will come across [3]. This is also known as developing one’s cultural metacognition. And, despite everyone’s goodwill and (hopefully) preparation, such instances will continue to occur at irregular intervals. The success of your international joint research project will, therefore, depend on how flexibly you can adjust to changing circumstances.

Other important questions to consider include the following: What is her ability to work well with other team members, particularly at a distance, across national boundaries? Is their work style complementary to yours, thus avoiding unnecessary conflicts? Does including the scientist or team you are targeting lend additional credibility and validity to the project? And if you are a non-native speaker of English (which is, after all, the lingua franca of scientific communication), is your potential partner well versed in writing and/or speaking the language? If all external signs are sufficiently positive to consider taking the next step, reaching agreement on a pilot project or a short-term feasibility study would be a prudent approach to assessing the extent to which your international collaborators’ abilities and working practices match your expectations and, subsequently, to potentially establishing a longer-term collaboration.

## Rule 3: Consider Practical Approaches to Establishing the Relationship

As active scientists at any level, from graduate student to senior professor, we encounter numerous opportunities to engage with international colleagues. Although the most obvious openings might arise through interactions at conferences or other meetings, either domestic or international, these are by no means the only suitable or even the most effective means of networking. Many university departments and research institutes run active visitors’ programs, often in the form of regular seminar series, which offer an ideal environment in which to meet and get to know international visitors. Conversely, you may have the opportunity to visit foreign institutes. If so, consider in advance to whom you would like to talk and whether you might have common interests that could potentially lead to joint research. And if you have moved to a new institution, do not forget the links you made with colleagues at your previous institute, particularly those who could offer complementary expertise that would benefit joint projects. In general, it is important to proactively pursue any collaborative opportunities.

In many cases, junior scientists will benefit from introductions made by their research supervisor or another local colleague, either in person or by email. And you could, of course, also approach a potential international collaborator yourself, but keep in mind that many people are very busy. It pays, therefore, to make your introduction interesting to your potential collaborator. In addition to asking for their time and/or resources, the most productive collaborations are established if you can offer a substantial benefit in return. This could be in the form of your time (particularly if you can take a leading role in the proposed project); your group’s resources, expertise, and external collaborations; or even additional introductions. In today’s interconnected world, networking is more important than ever, so by offering such an opportunity to your potential foreign collaborator, you may well have the edge!

## Rule 4: Define the Type of Collaboration You Want to Pursue

You may have considered setting up simple student exchanges, possibly through student co-supervision, but international collaborative projects often offer a multitude of additional opportunities to advance the overall research goals. It thus pays to consider, at an early stage, which type of collaboration you might want to pursue. There is no single answer to this important question, since this depends on the nature of the research question(s) to be tackled, the purpose and/or scope of the study, the extent and nature of the expertise you may require, administrative regulations or restrictions of the institutions involved, preferences of funding agencies, and possibly previous experience with your potential collaborators.

What is the overarching goal of the collaboration that cannot be achieved regionally or even domestically? Is it simply to provide interactions among researchers with different expertise, increased access to resources, or enhanced credibility? Are you after fresh perspectives to avoid “academic inbreeding,” or would you like to expand your research network? It is advisable to start relatively small, not least because larger international research collaborations often require substantial administrative resources, e.g., project management and financial reporting. You should be very confident that your international partners are indeed well matched before pursuing such large-scale opportunities. Many funding agencies, including national research councils and some nonprofit organizations, offer competitively awarded seed money for pilot projects or feasibility studies to explore whether the proposed international partners are indeed suitable to pursue more substantial research questions through subsequent joint efforts. Consider this your first step: short-term, fairly straightforward pilot projects are a great and low-risk way to get to know your international partner better and figure out whether you can work together productively and successfully.

## Rule 5: Clearly Define the Main Goals and Expected Outcomes

The golden rule in any collaborative context is to be as specific as you can be about the project’s goals during its development phase. Where international collaborations are concerned, it is particularly important to consider the conditions for success, and you should plan to evaluate all aspects of the collaboration rigorously. This is particularly important in the context of larger international collaborations, which are often closely audited externally, but you should adopt a positive attitude with respect to evaluation, by default. Clearly define the team members’ roles and responsibilities, and be prepared to offer significant time commitment yourself.

Fostering a high level of cooperative teamwork takes time and effort, particularly in international collaborations where cultural and language differences may prove challenging. Developing trust, collegiality, and a sense of fairness and accountability are at the basis of any successful research collaboration [2,4], irrespective of the team’s geographical distribution.

## Rule 6: Be Aware of the Most Important Obstacles to Establishing the Relationship

While establishing regional or domestic collaborations may already be challenging for a variety of mundane reasons, these difficulties might be amplified in an international context. For instance, conflicting research paradigms in different national settings, disagreements on conventions or standards of practice, as well as a lack of compliance with international research protocols may all affect the integrity of the joint research project. In addition, collaborators may not share the same professional jargon, or even speak the same working language sufficiently proficiently. One should certainly also be aware of cultural differences, even for collaborations between presumably similar research partners (such as scientists in the United States and the United Kingdom). While present-day electronic communication has made joint research across national borders viable and often highly successful, face-to-face meetings are arguably the most important vehicle by which to establish personal relationships with your international partner(s). Nothing can beat face-to-face discussions, ideally occurring at an early stage of your joint project, to overcome mundane obstacles such as a language barrier and cultural differences. This applies to both the workplace and social settings. Do not underestimate the importance of getting to know your international partner in a relaxed atmosphere; the best research collaborations are built on excellent personal relationships!

Differing opinions on the goals of and procedures pertaining to the research project, perhaps driven by such cultural differences or caused by disagreements regarding sharing time, work, data, or resources, may inadvertently affect the research collaboration. This particularly applies to awarding co-authorship on any publications resulting from the collaboration, as well as to proper attribution of credit for any of the team’s achievements. The most important keywords of relevance to international teamwork are transparency, openness, and careful planning. Above all, be prepared to be flexible while retaining ownership of the research collaboration.

## Rule 7: Discuss Dissemination Policies as well as Intellectual Property Rights at an Early Stage

In some research environments, principal investigators commonly include all members of their research group as co-authors, whereas this may be frowned upon elsewhere. This underscores the importance of reaching agreement on dissemination policies of the research outcomes as well as on authorship criteria. Ideally, this should be discussed at an early stage, well before disagreements may become an obstacle to the successful pursuit of the collaboration. Depending on your research focus and the expected outcomes, this is also the time to discuss and clarify the collaboration’s policies on commercialization and intellectual property rights. Note, however, that you will most likely be bound by the regulations established by your institution in this regard, so familiarize yourself with these boundary conditions.

Both the International Committee of Medical Journal Editors and the American Psychological Association have established well-regarded authorship criteria that are commonly adhered to by many international peer-reviewed journals, across disciplines [5,6]. It is recommended to consider the acceptable criteria for inclusion as (co-)author, the standard of acceptability regarding format and content of disseminated results, as well as the proper allocation of credit.

Establishing an effective dissemination policy requires reaching agreement on who will be authorized to speak on behalf of the collaboration, the target audience [7], and the nature and details of the information to be shared, both internally and externally [2]. The most important keywords in this context are trust and collegiality.

## Rule 8: Consider and Clarify the Extent to Which You Are Prepared to Share Resources

You should expect that senior researchers at high-profile international research institutes, particularly those possessing unique expertise, are busy scientists and project managers who receive numerous requests to embark on new collaborative projects every year. Your request will most likely fall on deaf ears if you do not offer anything in return, thus showing that you are a serious contender for your potential collaborator’s time and resources. It is, therefore, of the utmost importance to consider what you could offer your potential partner so that you will hit the ground running. Your contribution may include earmarked funding, personnel (such as shared students or postdoctoral researchers), proprietary data or analysis tools, access to specialized equipment, and/or novel ideas.

However, do not be disappointed if your request is turned down. There are numerous reasons why a potential collaborator may not be able to share their resources, including a need to protect preliminary work from criticism, claims related to discovery and priority (e.g., patents), intellectual property issues, safeguarding institutional or local investments, or confidential information (such as that related to peer review, human subjects, and medical data or in private, military, or forensic research [2]).

## Rule 9: Avoid Conflicts of Interest

While international collaborations can be highly rewarding, one should be acutely aware of the myriad conflicts of interest that might arise. In addition to issues related to (co-)authorship and intellectual property rights, the exchange of (medical) data between collaborators and countries can be a significant hurdle, and any data exchange policies need to be clarified at an early stage. In addition, some high-technology components are subject to US export controls under the International Traffic in Arms Regulations [8].

Your most important consideration should be to protect your reputation and your research integrity [9]. Full disclosure of any potential conflicts of interest is recommended, to funding agencies, institutions, collaborators, as well as journal editors. Avoid the appearance of biases, which may occur if such conflicts of interest come to the surface at a later stage. This could compromise the credibility of your study, even if nothing untoward happened. You could face claims of professional misconduct and, in extreme cases, your papers may be retracted.

## Rule 10: Be Aware of Potential Funding Opportunities

Different types of research require vastly different amounts and types of funding. Costs arising from research in an international context may include purchases of materials, travel expenses, publication and other dissemination charges, and personnel costs such as salaries of postdoctoral researchers or exchange students. Which of these costs apply to your collaboration depends on its nature. They can range from pilot funding to explore the viability of establishing a bilateral or multilateral collaboration to funding for exchanges of personnel (either as short- or long-term research projects) and large-scale multinational research projects, funded by the likes of the European Union (EU). Each of these will come with its own requirements and pressures.

In most cases, your main national funding agency is your first port of call. Standard research grants usually include an allowance for international expenditure and exchanges. Specific bilateral projects may be announced on an annual basis, while thematic calls for applications may include foreign investigators. Alternatively, consider submitting targeted funding applications to private, nonprofit foundations. These often focus on specific science, applications, or themes, and your joint research may well fit their briefs. Some of these foundations operate internationally and often target specific countries or regions, such as those in the developing world [10]. Your institution’s Research or International Offices are good entry points to start exploring these opportunities.

Finally, although there are many international or bilateral funding opportunities available for which your project may be eligible, it is not always easy to distinguish the wheat from the chaff. In the absence of a master list of funders, it is highly recommended to peruse the opportunities offered by various national research councils—many of which have established a presence well beyond their home nations’ borders, such as the British Council, the Alliance Française, the Humboldt Stiftung, and many others—as well as the EU’s offerings, which include the Marie Skłodowska-Curie actions and well-maintained lists of funding opportunities aimed at researchers from specific regions [11], including North America, China, and Japan, among others. And do not forget to check the websites of the embassy of the country where your potential research collaborator is based. You may be surprised by what the dedicated teams of science and technology councilors could offer you, although their deadlines may not come around regularly.
